# Linked seasonal outbreaks of *Salmonella* Typhimurium among passerine birds, domestic cats and humans, Sweden, 2009 to 2016

**DOI:** 10.2807/1560-7917.ES.2019.24.34.1900074

**Published:** 2019-08-22

**Authors:** Robert Söderlund, Cecilia Jernberg, Linda Trönnberg, Anna Pääjärvi, Erik Ågren, Elina Lahti

**Affiliations:** 1 National Veterinary Institute, Uppsala, Sweden; 2 Public Health Agency of Sweden, Solna, Sweden

**Keywords:** Salmonella, zoonosis, passerine birds, cats, high-throughput sequencing, MLVA, one health, molecular epidemiology

## Abstract

In 2016, an outbreak of *Salmonella* Typhimurium (STm) with multilocus variable-number tandem repeat analysis (MLVA) profiles historically associated with passerine birds (2-[11-15]-[3-4]-NA-212) occurred among passerines, cats and humans in Sweden. Our retrospective observational study investigated the outbreak and revisited historical data from 2009–16 to identify seasonality, phylogeography and other characteristics of this STm variant. Outbreak isolates were analysed by whole-genome single nucleotide polymorphism (SNP) typing. The number of notified cases of passerine-associated STm among passerines, cats and humans per month and county, and their MLVA profiles, were compared to birdwatchers’ counts of passerines. Seasonal trend decomposition and correlation analysis was performed. Outbreak isolates did not cluster by host on SNP level. Passerine-associated STm was seasonal for birds, cats and humans, with a peak in March. Cases and counts of passerines at bird feeders varied between years. The incidence of passerine-associated STm infections in humans was higher in the boreal north compared with the southern and capital regions, consistent with passerine population densities. Seasonal mass migration of passerines appears to cause STm outbreaks among cats certain years in Sweden, most likely via predation on weakened birds. Outbreaks among humans can follow, presumably caused by contact with cats or environmental contamination.

## Background

*Salmonella enterica* is a zoonotic and food-borne pathogen that is estimated to cause hundreds of millions of cases of disease worldwide every year [1]. In infected humans, *S. enterica* can cause diarrhoea, enteric fever or bacteraemia, depending on which subspecies and serovar causes the infection, as well as the susceptibility of the infected host [1]. Serovars occur in a spectrum ranging from host generalists, which can infect many animal species, to host specialists, which preferentially or only infect a single species [2]. Host adaptation can involve the acquisition of mobile genetic elements encoding new genetic traits [3,4], but also the loss of functions not needed in the preferred host or niche, e.g. metabolic capabilities [5]. Serovars that are specialists preferring particular animal hosts tend to cause infection among vulnerable human hosts like immunosuppressed people, young children and elderly people [2,6]. *S. enterica* subsp. *enterica* serovar Typhimurium (STm) is considered a predominantly generalist serovar, infecting a broad range of wild and domestic animal species [7], while also being the second most common source of human gastroenteritis caused by *Salmonella* in Europe [8]. However, certain variants within the serovar appear to be host specialists that are mostly found, for example, in pigeons [9], ducks [10] or hedgehogs [11].

Notable examples of host-specialist STm are the phage type DT 40/U277/NST variants, which have been reported as the cause of outbreaks of septicaemia and mortality in wild birds in several countries including Canada, Switzerland, Sweden, Norway and the United Kingdom (UK) [12-16]. Outbreaks tend to occur in late winter or early spring, most commonly among certain passerine species such as Eurasian siskins (*Carduelis spinus*), Eurasian bullfinches (*Pyrrhula pyrrhula*), common redpolls (*Carduelis flammea*) or greenfinches (*Carduelis chloris*). Emaciation and necrotic lesions in the oesophagus, crop, liver and spleen are frequently seen at necropsy [14,16]. The bacteria are generally present in blood, lung and liver samples, but absent from the intestines [14]. However, STm has also been found in cloacal swabs taken from apparently healthy passerines of the same species [14]. This suggests that the birds may serve as year-round hosts, carrying low levels of STm in their gastrointestinal tracts, with outbreaks occurring when birds are in poor condition and crowded at feeding locations in late winter or early spring. Infection among cats has also been reported in some of these outbreaks [15,16], and a study found STm phage type DT 40 in dead birds, in faecal samples from cats that had eaten birds and on the ground under a bird feeder in the same domestic garden area [17]. Infected cats can display inappetence, fever, vomiting and diarrhoea [15], but cats are also known to shed *Salmonella* while showing mild or no clinical signs of disease [18]. Given the exposure via cats and environmental contamination, these outbreaks pose a risk of causing human cases of salmonellosis. During an outbreak of STm DT 40/NST among passerines and cats in Värmland county, Sweden in 1999, four human cases of STm NST also occurred; two of these had sick cats in the household, while the other two had been feeding wild birds [15]. A recent study found that the same genotypes of STm occured among wild birds, cats and humans in the UK [19], further demonstrating the zoonotic potential of passerine-associated STm variants.

### Outbreak detection

In early 2016, several findings of dead passerine birds were reported by the Swedish public. At the same time, an uncommonly high number of STm-positive samples from domestic cats were analysed: 448 between January and March. Simultaneously, data from the national microbiological surveillance programme indicated an increase of STm with the multilocus variable-number tandem repeats analysis (MLVA) profile pattern 2-[11-15]-[3-4]-NA-212 among humans, with a total of 18 cases in the same period.

The overall number of domestic salmonellosis cases in Sweden is low; from 2009–16, there were 552–840 cases reported annually [20]. Salmonellosis is a notifiable disease in Sweden and all domestic isolates from humans are sent to the Public Health Agency (PHA)’s microbiological laboratory in Solna, Sweden for typing. The low number of cases and real-time typing of all isolates at the agency allow for a very sensitive surveillance system for outbreak detection. Results from MLVA typing showed matching profiles from passerines, cats and human cases during the same period.

The Swedish National Veterinary Institute (SVA) and the PHA initiated a collaborative project to investigate the outbreak and to review historical data on the occurrence of this passerine-associated variant of STm among passerines, cats and humans in Sweden.

## Methods

A retrospective, observational study was carried out to investigate the occurrence of a specific passerine-associated variant of STm among Swedish passerine birds, domestic cats and humans from 2009–16. The analysed data included observations from annual birdwatcher surveys; nationwide passive surveillance data regarding the occurrence of salmonellosis among Eurasian bullfinches, Eurasian siskins, common redpolls, cats and humans; molecular typing (MLVA) data from all sampled sources in Sweden (human, food, feed, domestic and wild animals); and whole genome sequencing typing data from passerine, cat and human isolates collected during the outbreak in 2016. Additional data from Statistics Sweden regarding regional rates of cat ownership and housing types was included to aid interpretation of the observations made.

### Birdwatcher surveys

Birdwatchers count and report the number of wild birds visiting private birdfeeders in Sweden the last weekend of January every year, an event organised by the Swedish Ornithological Society. Nationwide data on the numbers of common redpolls, Eurasian bullfinches and Eurasian siskins reported during this monitoring in the period 2009–16 were downloaded [21]. To compensate for the varying number of survey participants from year to year, these counts were normalised against the total number of birds counted from the top 30 species the same year.

### Samples and bacterial isolates

Routine MLVA analysis of all STm isolates from animal, food, feed and human sources was introduced in Sweden in 2009; therefore, the study period was set to 2009–16. Swedish authorities encourage the public to submit dead wild animals to the SVA for necropsy and ancillary laboratory analysis as part of the national wildlife disease surveillance programme. Passerine birds such as Eurasian bullfinches, Eurasian siskins and common redpolls that are found dead by the public are submitted on a volunteer basis. The study was limited to these three species, as they are the most commonly observed salmonellosis cases among wild birds in the SVA’s records. In instances where multiple birds were submitted from a single location and time, they were counted as a single observation.

A high proportion of Swedish cats have health insurance (estimated at 36% in 2014 [22]), and a veterinarian suspecting salmonellosis in an animal is obliged to perform an investigation. Therefore, many cats with signs of salmonellosis are investigated and sampled at veterinary clinics. Cat faecal samples from cats with suspected salmonellosis are submitted to the SVA’s laboratory for *Salmonella* analysis. Presumptive *Salmonella* isolates are also submitted to the SVA from other Swedish laboratories for mandatory confirmation of possible cases of salmonellosis in cats.

A subset of the isolates investigated in this study was not fully serotyped, with an O4+ status considered sufficient to assume the isolate to be STm. Not all isolates from cats and wild birds were typed by MLVA because of volume caps that are implemented for practical and economic reasons in response to the high number of very similar profiles that are generated in years when many cases occur.

Human *Salmonella* spp. isolates from domestic cases are routinely submitted to the PHA by clinical microbiological laboratories for typing. All human STm isolates from 2009–16 were analysed by MLVA. The human case definition for the present study was domestic infection by STm with a MLVA profile matching the passerine-associated pattern 2-[11-15]-[3-4]-NA-212.

### Molecular characterisation

Phage typing has been replaced by MLVA for typing of STm isolates in Sweden. MLVA was performed according to the European Centre for Disease Prevention and Control’s standard protocol [23]. Whole genome sequencing was performed on isolates from 15 passerines (2 Eurasian siskins, 4 common redpolls and 9 Eurasian bullfinches), 15 cats and 10 humans during the 2016 outbreak. A convenience sample of isolates from different parts of the country was included for each host category to reveal any major regional differences. DNA was extracted using the Blood & Tissue Kit protocol on a BioRobot EZ1 (Qiagen, Hilden, Germany), with libraries subsequently prepared using the Nextera XT kit (Illumina, San Diego, California, United States). Sequencing was performed on a MiSeq instrument (Illumina) as paired-end 2 x 250 bp reads, with all isolates sequenced to > 25 x depth. Single nucleotide polymorphism typing was performed by mapping reads corresponding to ca 25 x–100 x coverage for each isolate to the complete STm LT2 genome sequence [24] using Bowtie 2 2.2.7 [25], and calling SNPs with SAMtools 1.3.1 [26]. SNPs were filtered, requiring a single variant allele for each included variable site, an overall quality > 100 for each variable site, and for each variable site to be present and have a quality > 25 in all isolates. Sites with a conflicting genotype call with a quality of > 10% of the primary call quality were excluded. The relationship between isolates was visualised using the neighbor-net algorithm in SplitsTree 4.14.4 [27]. All sequence data were uploaded to the European nucleotide archive (ENA) and are available under project accession number PRJEB27180.

### Population and geographical data

Counties were classified into three zones based on biogeography [28] to relate the occurrence of passerine-associated STm cases with the ecology of the investigated bird species: (i) the boreal zone, characterised by coniferous and birch forests (counties BD, AC, Z, Y, X, W, S and T in northern and central Sweden); (ii) the boreo-nemoral zone, characterised by mixed deciduous and coniferous forests and a generally lower overall forest cover (counties U, C, D, O, E, F, G, H, I and K in central and southern Sweden) and (iii) the nemoral zone, characterised by deciduous forests and lower overall forest cover (counties N and M in the southernmost part of Sweden). The capital region of Stockholm county (AB), located in the boreo-nemoral zone, was analysed as a separate category (See Supplementary Table S1 for county codes). The official county-level human population for 2016 was retrieved from Statistics Sweden [29]. Supplementary data for 2016 regarding households living in apartment buildings vs detached or semi-detached houses, at the county level, were available from the Statistics Sweden online database [29] and were used as a proxy to identify possible differences in exposure to STm from wild birds via access to a garden or feeding birds. Data on household cat ownership were available by Nomenclature of Territorial Units for Statistics (NUTS) 2 county groups from a study performed in 2012, also from Statistics Sweden [30]. Age, sex and county of residence for human cases was retrieved from SmiNet, the Swedish electronic notification system for notifiable communicable diseases. No data on the severity of symptoms or underlying conditions were available.

### Data analysis

Associations between year-to-year microbiological and birdwatcher survey data were investigated using Spearman’s rank correlation test in R version 3.3.1 (R Foundation, Vienna, Austria), with the alternative hypothesis that the true rho (ρ) was not equal to 0. Any p value < 0.05 was considered significant; 95% confidence intervals (CIs) for incidence measurements were calculated using the Agresti-Coull method [31] implemented in the binom package in R version 3.3.1. Differences were considered significant if CIs did not overlap. Seasonal trend decomposition of the occurrence of STm among cats, birds and humans was performed using the stl function in R version 3.3.1, dividing the observed variation into seasonal, long-term trend and random components.

### Ethical statement

Data on human cases were collected as part of the national surveillance of salmonellosis under the Swedish Communicable Diseases Act (SFS: 2004:168). All data were anonymised and cannot be inferred directly or indirectly to a person. Guidelines on animal ethics and welfare were followed. According to national legislation, ethical permission is not needed when animals are sampled at veterinary clinics for diagnostic purposes. Persons submitting samples to the SVA give their consent on the referral form regarding the use of the submitted material for research purposes. Wild birds were sampled within the general disease surveillance of found dead birds, where approval from an ethical review committee is not required.

## Results

### Birdwatcher counts of selected passerine species at bird feeders

**Figure 1:**
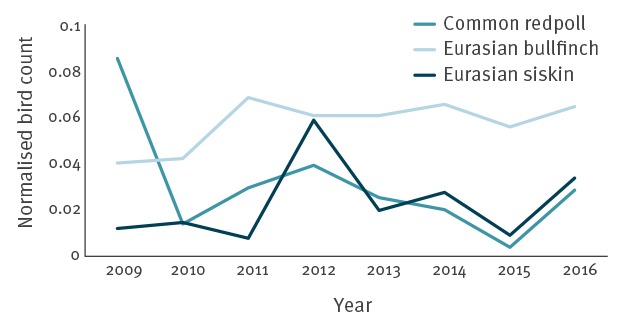
Normalised counts of selected passerine species at bird feeders, Sweden, last weekend of January 2009–2016

Birdwatcher observation data analysed in the present study showed a marked year-to-year variability in the number of Eurasian siskins and common redpolls (Figure 1). Notably, 4,117 common redpolls and 9,456 Eurasian siskins were counted in 2015, increasing to 23,041 and 27,242, respectively, in the STm outbreak year of 2016. A surge of common redpolls also occurred in 2009 and of Eurasian siskins in 2012. The number of Eurasian bullfinches counted was less variable. A total of 874,483 visits from individual birds of the species of interest were counted, while the total count in the normalisation dataset was 9,299,153 birds.

### *Salmonella* Typhimurium among passerines and cats

STm isolates from 72 Eurasian bullfinches, Eurasian siskins and common redpolls, submitted from 2009–16, were included in the study (Supplementary Table S2). The outbreak year of 2016 coincided with the highest number of birds diagnosed with STm. The number of found dead birds submitted per year varied between none and 27, with an average of nine per year.

A total of 1,165 index isolates of STm from cats, submitted by veterinarians from 2009–16 for *Salmonella* analysis or for confirmation of presumptive isolates, were included in the study. The number of isolates collected per year varied between seven and 487, with an average of 146 per year. Of the 1,165 isolates, 805 (69%) were not fully serotyped, as their O4+ status was considered sufficient to assume the isolate to be a likely STm.

Of the passerine and cat isolates, a subset of 54 (75%) and 116 (10%) isolates, respectively, were analysed by MLVA.

### Multilocus variable-number tandem repeat analysis comparison of isolates from passerines, cats and humans

MLVA profiles were homogenous for the 54 passerine isolates, all of which matched the pattern 2-[11-15]-[3-4]-NA-212 (Table 1), referred to herein as ‘passerine-associated’. Of the 116 cat isolates, 106 had profiles matching those of the passerines, six were single-locus variants of multiple passerine profiles and four differed at three loci or more from all passerine profiles. In contrast, isolates from domestic cases of STm among humans had a rich variety of MLVA profiles and were only included in the study if they matched the passerine-associated MLVA pattern 2-[11-15]-[3-4]-NA-212, which was selected based on the variants that were observed among passerines. This pattern was found in 86 human isolates from patients with a mean age of 40 years and a median age of 49 years, of which 52% (n = 45) were women. For humans, as for the passerines and cats, 2-13-3-NA-212 was the most common passerine-associated MLVA profile, followed by 2-12-3-NA-212 (Table 1). A database search for the same profile among STm from other sources in Sweden—including food, animal feed, and wild and domestic animals—produced very few hits, mostly isolates from other passerine birds like great tits and greenfinches or from predators like red foxes and birds of prey (data not shown). All findings of STm in food, feed, domestic animals and other wildlife were typed by MLVA during the study period.

**Table 1:** MLVA profiles of *Salmonella* Typhimurium from cats, passerines and matching isolates from domestic human salmonellosis cases, Sweden, 2009–2016

MLVA profile	Number of passerines	Number of cats	Number of humans
2-13-3-NA-212	20	47	47
2-12-3-NA-212	16	29	11
2-11-3-NA-212	9	14	9
2-14-3-NA-212	4	9	11
2-13-4-NA-212	2	4	7
2-12-4-NA-212	2	1	0
2-15-3-NA-212	1	2	0
2-14-4-NA-212	0	0	1
2-10-3-NA-212	0	4	ND
3-13-10-NA-211	0	2	ND
2-13-2-NA-212	0	1	ND
2-12-NA-NA-212	0	1	ND
3-20-15-9-309	0	1	ND
3-18-NA-NA-211	0	1	ND
**Total**	**54**	**116**	**86**

MLVA: multilocus variable-number tandem repeat analysis; ND: not done.

### Passerine-associated *Salmonella* Typhimurium as a cause of human salmonellosis

**Figure 2:**
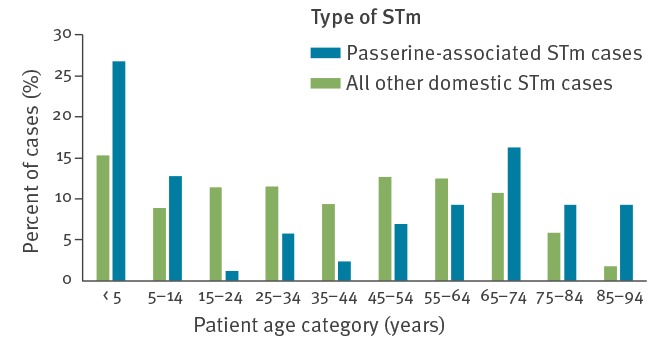
Age distribution of domestic cases of *Salmonella* Typhimurium with (n = 86) and without (n = 1,281) MLVA profiles consistent with passerines as the ultimate source of infection, Sweden, 2009–2016

MLVA: multilocus variable-number tandem repeat analysis; STm: *Salmonella* Typhimurium.

Using the passerine-associated MLVA profiles as selection criteria, 86 cases of human salmonellosis for which passerines or cats are probable sources were identified in Sweden in 2009–16. This corresponds to 6% of the human cases of STm infection contracted within the country. The number of passerine-associated cases varied between three and 25 per year, with an average of 11 per year; young children (< 5 years) and elderly people (≥ 60 years) were overrepresented among these cases, compared with all domestic STm cases (Figure 2). The largest number of cases occurred in 2016, when 25 isolates were identified as passerine associated. Specifically, the 2-13-3-NA-212 profile dominated, with 12 cases reported in January–March 2016. Of these 12 cases, five were children < 5 years of age and five were > 60 years of age. Local reports indicated that a number of cases had contact with cats (data not shown).

‘Passerine-associated’ STm refers to variants with the MLVA pattern 2-[11-15]-[3-4]-NA-212, which can indicate passerines as a probable source.

**Figure 3:**
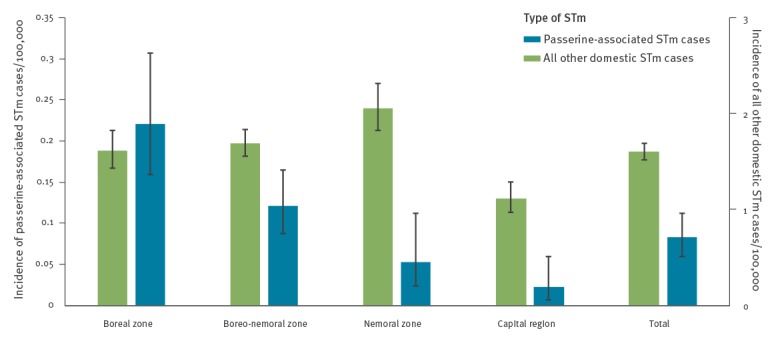
Reported incidence of domestic human *Salmonella* Typhimurium cases with (n = 86) and without (n = 1,281) MLVA profiles consistent with passerines as the source of infection, by biogeographical zone, Sweden, 2009–2016

MLVA: multilocus variable-number tandem repeat analysis; STm: *Salmonella* Typhimurium.

A comparison of the incidence of passerine-associated STm between different biogeographical zones in Sweden in 2009–16 revealed differences. The incidence was significantly higher in the northern boreal zone (0.22/100,000 inhabitants) compared with the nemoral zone (0.05/100,000) and the capital region of Stockholm county (0.02/100,000) (Figure 3). No significant differences between the zones were observed when comparing the incidence of domestic STm cases caused by all other types, except for Stockholm county, which has a significantly lower overall incidence (Figure 3).

‘Passerine-associated’ STm refers to variants with the MLVA pattern 2-[11-15]-[3-4]-NA-212, which can indicate passerines as a probable source.

### Whole genome sequencing investigation of the 2016 outbreak

**Figure 4:**
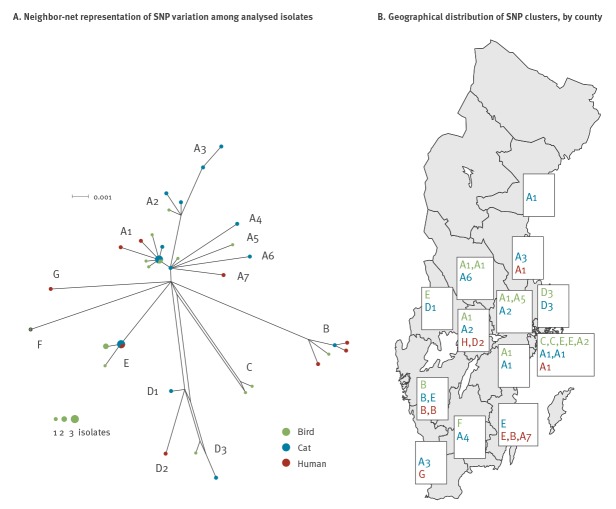
(A) Whole-genome SNP analysis of of *Salmonella* Typhimurium isolates from an outbreak affecting birds, cats and humans and (B) county-level phylogeography, Sweden, 2009–2016 (n = 40)

The 2016 outbreak among passerines, cats and humans in Sweden was caused by a genetically homogenous group of strains, as indicated by the MLVA analysis, but there was substantial variation between outbreak isolates on the whole-genome SNP level (Figure 4a). The outbreak isolates did not cluster by host, with most of the main clades of outbreak isolates containing representatives from passerines, cats and humans. A single human isolate, the earliest in the year among the 10 analysed, differed more substantially from the other outbreak isolates in terms of SNP variation; however, it was consistent with the outbreak in terms of MLVA profile (cluster H, not shown in the network in Figure 4a). The 40 outbreak isolates investigated were from 13 counties (Figure 4b). There were several instances of the same genotype occurring in multiple host types in the same county, but otherwise a limited regionality of genotypes.

Cluster H is not shown in the network, as it was atypical and did not cluster with the others.

### Year-to-year variation and seasonality

**Figure 5:**
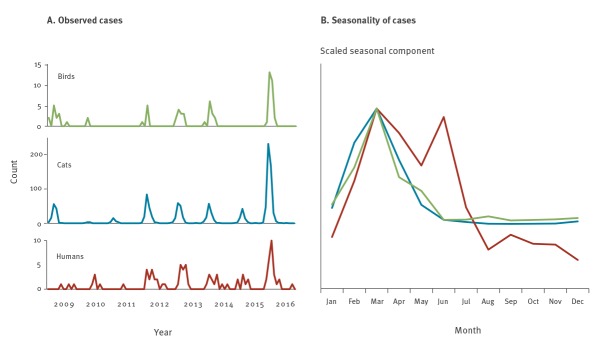
(A) Year-to-year variation and (B) seasonality of presumed passerine-associated *Salmonella* Typhimurium among passerines (n = 72), cats (n = 1,165) and humans (n = 86), Sweden, 2009–2016

Comparing data on the number of cases of passerine-associated STm during the 2009–16 period revealed variation between years for passerines, birds and humans. The year 2016 was a record high for all three host types (Figure 5), but was preceded by a very low number of notified cases in 2015. The variation between years was positively correlated for all three host types (Spearman’s rho 0.5–0.9), with a significant correlation between humans and cats (Table 2). The birdwatcher counts of relevant passerine species was also positively correlated with STm in all three host types (Spearman’s rho 0.4–0.7), but not significantly so. As evident in Figure 1 and Figure 5, a short-range mass migration event, referred to as an ‘irruption’, of common redpolls in northern Sweden in 2009 coincided with an outbreak among cats, but few or no human cases, reducing the overall strength of correlations. With 2009 excluded from the analysis, the annual number of human cases was significantly correlated with both cat and passerine cases. Combining data from all 8 years by seasonal decomposition analysis showed a marked seasonality with passerine-associated STm the most common among all three host types in March (Figure 5, Supplementary Figure S3). Very few cases were observed among passerines or cats at any time other than the first 4 months of each year, while human cases continued into early summer. The overall number of domestic cases of STm among humans, regardless of MLVA profile, varied between 552–840 during the study period (Supplementary Table S4).

**Table 2:** Correlations^a^ in year-to-year variation in the number of cases of presumed passerine-associated *Salmonella* Typhimurium among passerines, cats and humans, and the number of selected passerine species counted by birdwatchers, Sweden, 2009–2016

Correlation	PA STm in passerines	PA STm in cats	PA STm in humans
PA STm in passerines	NA	ρ = 0.7, p = 0.05	ρ = 0.5, p = 0.2
PA STm in cats	ρ = 0.7, p = 0.05	NA	ρ = 0.9, p = 0.005
Passerine count	ρ = 0.7, p = 0.06	ρ = 0.7, p = 0.06	ρ = 0.4, p = 0.4

NA: not applicable; PA: passerine-associated; STm: *Salmonella* Typhimurium.

^a^ Spearman’s rank correlation test.

### Outbreak control measures

The Swedish national authorities launched a public information campaign at the time of the 2016 outbreak. Press releases and web notices highlighted the importance of hygiene when handling bird feeders and cat litter boxes, cleaning the area under bird feeders and keeping cats showing signs of disease away from children. This information was picked up by several national and regional media outlets and raised public awareness. While the human cases were comparatively few, the same precautions can also be beneficial for preventing the spread of, for example, psittacosis and avian trichomoniasis.

## Discussion

Most salmonellosis cases in Sweden are related to travel abroad or contaminated food, but the occurrence of cases associated with cats in the early months of the year has been anecdotally known for many years [15]. We have used nationwide data from multiple sources, covering the period 2009–16, to show that multi-species outbreaks of passerine-associated STm occur among passerine birds, domestic cats and humans in the early months of certain years, possibly triggered by fluctuations in the passerine population and mass migration events. Birdwatcher data confirm that passerines in Sweden seek out human habitations to feed in large numbers in certain years, resulting in varying levels of exposure to passerine-associated STm from year to year for both domestic cats and humans. The underlying drivers of this phenomenon are likely complex. Many tree species produce large seed crops intermittently, with low or no production during the interim years; these cycles can be synchronous over large geographical areas, thereby affecting the ecology of the animal species that feed on the tree seeds [32]. For example, spruce and birch seed production is known to influence the population size and winter movements of common redpolls and Eurasian siskins, respectively, with years of low seeding triggering irruptions and increased mortality [33-35]. Other factors, such as the seed crop the previous year [36], the weather [37] and the availability of alternative food sources (like the seeds of annual plants [15]) have also been thought to trigger irruptive migration among passerines. Predation on weakened birds with possible septicaemia, e.g. around bird feeders, is the most likely route of infection for cats [15,17], presumably exposing the cat to a high infectious dose.

The human incidence of passerine-associated STm was significantly higher in the boreal northern and central parts of Sweden. While this could be an artefact of the limited number of observations and years of sampling, as well as other uncertainties related to under-reporting of salmonellosis in humans, it is biologically plausible, as middle to northern Sweden is richer in spruce and birch forest habitats and therefore supports larger populations of the relevant passerine species [35]. We also note a low incidence in the capital region of Stockholm county. Fewer households in Stockholm county than nationwide have one or more cats (10% vs 17%), and more households live in apartment buildings (59% vs 41%). Thus, it is likely that a lower proportion of the population in Stockholm county interacts with a garden bird feeder or an outdoor cat, compared with other counties in the boreo-nemoral zone, presumably leading to less exposure to passerine-associated STm.

In Sweden, passerine-associated STm among humans seems to more commonly afflict young children and elderly people. It is possible that these groups have a higher exposure to outdoor cats, bird feeders and garden environments. In addition, passerine-associated STm appears to be host biased and lacks the large virulence plasmid found in many other strains of STm [19], traits associated with a lower risk of severe infection in humans [2,4]. Healthy adults may therefore hypothetically be less vulnerable to infection with this variant of STm, compared with other variants.

We observed that passerine-associated STm was strongly seasonal in passerines, cats and humans. All three host types experienced a peak in the early months of the year, particularly in March, with human cases continuing to occur during early summer, when cases among cats and birds declined. It is conceivable that asymptomatic birds or persistent environmental contamination continue to cause these human infections later in the season. The peak of this variant of STm is in contrast with other domestic STm infections and salmonellosis in general in Sweden, which peak in late summer. Both MLVA and whole genome sequencing confirmed the link between the different hosts of the pathogen, although, as expected, sequencing data were found to be more informative. The 2016 outbreak was not clonal, consistent with an outbreak caused by environmental triggers acting on multiple sources of infection simultaneously—in this case, populations of passerines in different areas, as opposed to the highly clonal outbreak strains frequently found in single-source, e.g. food-borne, outbreaks.

The presented data should be interpreted with caution, as it is largely based on passive clinical surveillance. Continued observation over longer periods is therefore warranted. However, although we are far from understanding this complex phenomenon, we propose that the observation of high numbers of passerines like Eurasian siskins and common redpolls in winter can be used as an early warning of an increased risk of outbreaks of salmonellosis among cats and humans. The common redpoll and Eurasian siskin are resident in much of northern Eurasia as well as further south, e.g. in central Europe and the Alps [38], and occur seasonally in most of mainland Europe [38]. The Eurasian bullfinch is resident in most of Europe [38]. As previously mentioned, outbreaks of salmonellosis among these birds have been reported from several European countries [13-17]. In North America, the common redpoll co-occurs with the pine siskin (*Spinus pinus*), a close relative of the Eurasian siskin [38], with both species experiencing periodic outbreaks of salmonellosis [12]. Our results are therefore likely to have implications for public and animal health outside our study area of Sweden. Typing methodologies like MLVA and whole genome sequencing have facilitated data exchange between veterinarian and public health sectors, as well as the discovery of discrete lineages of pathogens, thereby improving the capacity to trace zoonotic spread of bacteria. Continued use of such methods, as well as retrospective analysis of historical isolates and international data sharing, is likely to reveal more host-adapted lineages of STm and epidemiological links between animals and humans in the years to come.

## Supplementary Data

200000 Supplementary Material
