# Supplementary material for: Linked seasonal outbreaks of Salmonella Typhimurium among passerine birds, domestic cats and humans, Sweden, 2009 to 2016
Source: Euro Surveill. 2019 Aug 22;24(34):1900074. doi: 10.2807/1560-7917.ES.2019.24.34.1900074 (PMC6712933; doi:10.2807/1560-7917.ES.2019.24.34.1900074)
Supplement: Supplementary Material [file 19-00074_SODERLUND_Supplement.pdf]

Supplementary material

This supplementary material is hosted by *Eurosurveillance* as supporting information alongside the article "**Linked springtime outbreaks of Salmonella Typhimurium among passerine birds, domestic cats and humans in Sweden, 2009-2016**" on behalf of the authors who remain responsible for the accuracy and appropriateness of the content. The same standards for ethics, copyright, attributions and permissions as for the article apply. *Eurosurveillance* is not responsible for the maintenance of any links or email addresses provided therein.

Supplementary Table S1 – County codes

Boreal

BD – Norrbottens län
AC – Västerbottens län
Z – Jämtlands län
Y – Västernorrlands län
X – Gävleborgs län
W – Kopparbergs län / Dalarnas län
S – Värmlands län
T – Örebro län

Nemo-boreal

C – Uppsala län
U – Västmanlands län
AB – Stockholms län (Capital region)
D – Södermanlands län
O –Västra Götalands län
E – Östergötlands län
F – Jönköpings län
H – Kalmar län
I – Gotlands län
G – Kronobergs län
K – Blekinge län

Nemoral

N – Hallands län
M –Skåne län

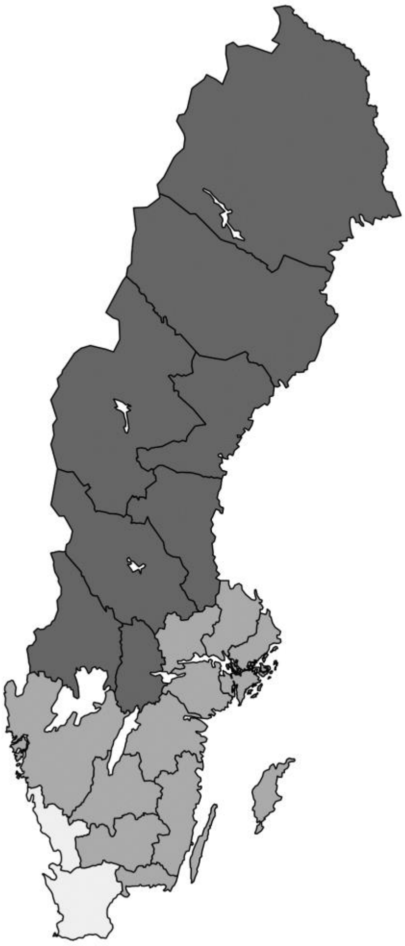

Supplementary Table S2 – Passerines with STm 2009-2016

Zone	2009 (B/S/R)	2010	2011	2012	2013	2014	2015	2016	Total
Boreal	(0/0/8)	0	0	0	(5/0/5)	(2/0/0)	0	(5/2/0)	(12/2/13)
Nemo-boreal	(0/0/3)	(0/2/0)	0	(0/4/0)	(1/0/0)	(5/5/0)	0	(10/1/0)	(16/12/3)
Nemoral	(0/0/1)	0	0	(1/0/0)	0	0	0	0	(1/0/1)
Capital	(0/0/1)	0	0	(0/0/1)	(0/1/0)	0	0	(2/2/5)	(2/3/7)
**Total**	(0/0/13)	(0/2/0)	0	(1/4/1)	(6/1/5)	(7/5/0)	0	(17/5/5)	(31/17/24)

Dead passerines (B bullfinches, S siskins, R redpolls) submitted by the public and diagnosed with STm in Sweden by biogeographical zone and year, 2009-2016. The capital region of Stockholm County, located in the nemo-boreal zone, is shown separately.

Supplementary Figure S3 – Seasonal decomposition

Seasonal decomposition of monthly data on the number of cases of passerine type STm among passerines (black) cats (blue) and humans (red) performed using the *stl* function in R 3.3.1, with each plot consisting of four panels showing (from the top down) the observations, seasonal component, trend component and remainder.

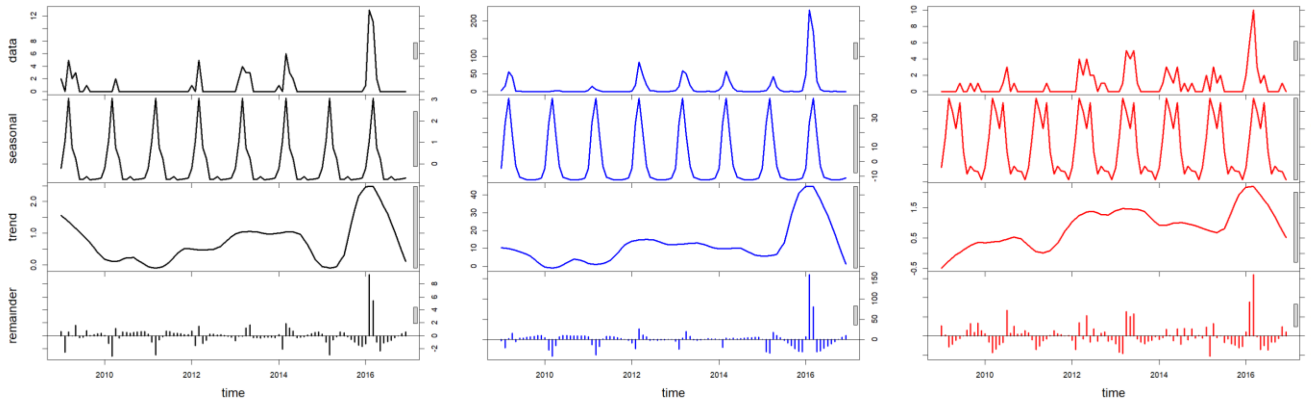

Supplementary Table S4 – Domestic cases of salmonellosis in Sweden 2009-2016

Number of cases of domestically acquired Salmonella infection in Sweden annually 2009-2016, and the corresponding incidence expressed as cases per 100,000 population.

**Year**	**2009**	**2010**	**2011**	**2012**	**2013**	**2014**	**2015**	**2016**
Number of cases	597	840	785	665	654	552	691	648
Incidence (cases per 100k)	6.4	8.9	8.3	7.0	6.8	5.7	7.0	6.5
